# Advances in the Drug Development and Quality Evaluation Principles of Oncolytic Herpes Simplex Virus

**DOI:** 10.3390/v17040581

**Published:** 2025-04-18

**Authors:** Basma Eid Abdullah Ghorab, Tongtan Liu, Min Ying, Ping Wang, Meirong Qin, Jiayong Xing, Huadong Wang, Fuqiang Xu

**Affiliations:** 1 Shenzhen Key Laboratory of Viral Vectors for Biomedicine, Shenzhen-Hong Kong Institute of Brain Science, Shenzhen Institutes of Advanced Technology, Chinese Academy of Sciences, Shenzhen 518055, China; basmagorab125@gmail.com (B.E.A.G.); tt.liu2@siat.ac.cn (T.L.); xingjiayong24@mails.ucas.ac.cn (J.X.); 2 NMPA Key Laboratory for Research and Evaluation of Viral Vector Technology in Cell and Gene Therapy Medicinal Products, CAS Key Laboratory of Brain Connectome and Manipulation, The Brain Cognition and Brain Disease Institute, Shenzhen Institutes of Advanced Technology, Chinese Academy of Sciences, Shenzhen 518055, China; yingmin@alumni.hust.edu.cn; 3 Key Laboratory of Quality Control Technology for Virus-Based Therapeutics, Guangdong Provincial Medical Products Administration, Guangdong Provincial Key Laboratory of Viral Biotechnology and Application, Shenzhen Institutes of Advanced Technology, Chinese Academy of Sciences, Shenzhen 518055, China; 4 University of Chinese Academy of Sciences, Beijing 100049, China; 5 Department of Anesthesiology, Second Affiliated Hospital, Third Military Medical University, Chongqing 400037, China; 6 Shenzhen Institute for Drug Control, Shenzhen 518057, China; wangping662@sina.com (P.W.); szqinmeirong@sina.com (M.Q.); 7 State Key Laboratory of Magnetic Resonance and Atomic and Molecular Physics, Innovation Academy for Precision Measurement Science and Technology, Chinese Academy of Sciences, Wuhan 430071, China; 8 Center for Excellence in Brain Science and Intelligence Technology, Chinese Academy of Sciences, Shanghai 200031, China; 9 Wuhan National Laboratory for Optoelectronics, Huazhong University of Science and Technology, Wuhan 430074, China

**Keywords:** oncolytic virus, herpes simplex virus, drug design, combination therapy, quality control, evaluation principles

## Abstract

Oncolytic herpes simplex virus (oHSV) represents a promising therapeutic approach to treating cancers by virtue of its selective replication in and lysis of tumor cells, with stimulation of host antitumor immunity. At present, four OV drugs have been approved for the treatment of cancers worldwide, two of which are oHSV drugs that have received extensive attention, known as T-VEC and Delytact. This review discusses the history, mechanism of action, clinical development, quality control, and evaluation principles of oHSV products, including viral species and genetic modifications that have improved these products’ therapeutic potential, limitations, and future directions. Integration of oHSVs with immunotherapeutic agents and conventional therapies has a promising future in the field of treatment of malignant tumors. Although much progress has been achieved, there is still much work to be done regarding the optimization of treatment protocols and the quality control of oncolytic virus drugs. The approval of various oncolytic virus therapies underlines their clinical relevance, safety, and efficacy, thereby paving the way for further research aimed at overcoming the existing limitations and enhancing patient responses.

## 1. Introduction

In the late 18th century, reports about the inhibiting effect of viral infections on cancer were akin to a beacon of light, encouraging scientists to focus more on oncolytic viruses [1,2]. Their promotion of tumor regression through discriminative replication in tumor cells, induction of tumor cell death, and promotion of host antitumor immunity made OVs a novel class of therapeutic cancer agents [3,4,5]. From the 1980s to now, many versions of oHSVs have been developed and tested in preclinical and clinical trials [6]. Four OVs have received approval worldwide thus far to treat advanced malignancies. The first was Rigvir, an RNA virus approved for the treatment of melanoma in Latvia in 2004. It was developed from the native ECHO-7 strain of a picornavirus [7,8,9,10]. Then, in 2005, China authorized the use of H101, a genetically engineered adenovirus, together with cytotoxic chemotherapy, to treat nasopharyngeal cancer [11,12]. The U.S. Food and Drug Administration (FDA) authorized an attenuated herpes simplex virus type 1 in 2015, which encodes granulocyte–macrophage colony-stimulating factor (GM-CSF), talimogene laherparepvec (T-VEC), as a local therapy for patients suffering from recurrent melanoma, which specifically treats unresectable cutaneous, subcutaneous, and nodal lesions [13,14,15,16,17,18,19,20,21,22,23]. In 2021, Daiichi Sankyo announced that the oncolytic viral therapy Delytact (teserpaturev, or G47Δ) was approved by the Japanese Ministry of Health, Labor, and Welfare and was officially launched for the treatment of malignant gliomas in Japan [24].

As the genetic heterogeneity of tumors can lead to the selection of phenotypes resistant to single-agent therapies, recently, immunotherapeutic agents such as immune checkpoint inhibitors, histone deacetylase (HDAC) inhibitors, MAPK pathway inhibitors, and TGFβ inhibitors have been used in combination with oHSVs, inducing antitumor immune responses and demonstrating a higher efficacy in targeting cancer cells [6,25,26,27]. The quality control and evaluation of OVs are essential principles and guidelines for validating and improving the efficacy, safety, and consistency of OVs for therapeutic application. There are also potential risks in dealing with viruses; thus, essential quality control principles must be strictly followed to avoid any transmission, infection, or epidemic disaster [28,29]. In this review, we focus on summarizing the historical context, mechanism of action, design, preclinical and clinical trials, combinational therapies of oHSV, quality control and evaluation principles of OVs, limitations, strategies to overcome these limitations, and future directions of oHSV development.

## 2. Viruses: From Toxicity to Oncolysis

In the late 18th century, it was observed that some viruses could be used to target and kill cancer cells. For example, the influenza virus showed high effectiveness in cancer inhibition in cancer patients with influenza infection [1,2,30,31,32,33,34]. In a study conducted in 1922, the vaccinia virus showed tumor inhibition in animal models. Furthermore, the Russian Far East encephalitis virus showed complete inhibition of mouse sarcoma 180. In 1950, the National Cancer Institute conducted experiments using adenoviruses, which represented the first clinical trials of viral oncolysis. However, despite the groundbreaking works, their serious side effects and minimal effectiveness resulted in a reduced focus on research on oncolytic viral treatment over the following decades. Nonetheless, progress in molecular biology and genetic engineering resulted in a renewal in research on oncolytic viral therapies in the late 20th century [2,35]. Scientists started to focus on the genetically engineered oncolytic herpes simplex virus (oHSV), which was modified to provide greater specificity and efficacy in infecting and killing tumor cells while avoiding normal tissue. Since its inception, it has demonstrated appealing and practical qualities, supported by extensive studies and recent advancements [2,36].

## 3. Mechanism of Action

The antitumor activity of oHSV is mediated through several interconnected processes. The structure of oncolytic herpes simplex virus represents the key to its ability to infect tumor cells and avoid normal tissue. Oncolytic HSV is a dsDNA virus that contains 152 kilobases, with about 30 kilobases transcribing non-essential genes [37,38,39,40,41,42,43]. Genetic modifications of non-essential genes provide greater specificity and efficacy in infecting and killing tumor cells while avoiding normal tissues [37]. One of these modifications is the thymidine kinase (TK) gene deletion. The TK gene of HSV-1 is a gene that allows HSV-1 to replicate in non-dividing cells. When wild-type HSV-1 infects normal cells, HSV-1 depends on its TK gene to replicate, as normal cells do not have sufficient TK proteins. HSV-1 can replicate within cancer cells independently of its TK gene, as cancer cells produce extra TK. The deletion of the TK gene improves oHSV’s ability to target cancer cells (such as glioma) while avoiding normal cells [44,45]. The ICP34.5-encoding gene, which is considered an important neurovirulence gene in wild-type HSV-1, is also one of these non-essential genes [46]. When wild-type HSV-1 infects cells, the cells try to defend themselves by activating protein kinase R (PKR), which is a protein responsible for preventing viral replication. However, the presence of the ICP34.5-encoding gene allows the virus to overcome PKR and replicate in cells [47]. Deletion of the ICP34.5-encoding gene prevents oHSV from infecting normal cells. As cancer cells have impaired PKR, oHSV, which lacks the ICP34.5-encoding gene, can selectively infect and replicate within tumor cells, leading to tumor cell death [37,48,49]. Furthermore, the US11 gene is known as a late viral protein that enhances the ability of HSV to replicate inside cells through binding and suppressing PKR. Besides the ICP34.5-encoding gene deletion, the US11 gene can be modified through shifting it under an early promoter to accelerate its expression. This modification improves oHSV’s ability to replicate inside tumor cells [50]. Furthermore, the deletion of the ICP47-encoding gene, which is a protein that helps HSV-1 to escape from the immune system through binding with TAP proteins, is considered to be another modification that improves the ability of oHSV to kill cancer cells [51]. Additionally, because the ICP47-encoding gene region blocks the early expression of the US11 gene, replacing the US11 gene under an early promoter is almost equivalent to the ICP47-encoding gene deletion. This modification allows the US11 gene to express earlier, leading to an enhancement of oHSV’s ability to replicate within cancer cells [52]. Additionally, *ICP6* is an element of the viral replication system and allows HSV-1 to replicate its genomic substance [37]. *ICP6* deletion limits viral replication to only dividing cells, such as tumor cells, and avoids quiescent healthy tissues [37,53].

Immune response activation represents another mechanism of oHSV in tumors. When oHSV infects tumor cells, it replicates, causing tumor cell lysis, which initiates antiviral and antitumor immunity [37,54,55,56,57,58]. The antiviral response leads to tumor cell death, while antitumor immunity induces immune cells to attack cancer cells. Immunogenic cell death (ICD) is a key component of immune stimulation, which results from oHSV infection in tumor cells and produces damage-associated molecular patterns (DAMPs) [37,59,60,61]. These components help the immune system identify and recognize tumor cells. DAMPs act as immature dendritic cell (DC) activators, resulting in DC maturation. The mature DCs then act as presenters of tumor antigens to T cells, allowing T cells to attack tumor cells. Production of CC-chemokine ligand 4 (CCL4) is also a key response to immune stimulation and attracts immune cells to the tumor microenvironment. Pattern recognition receptors facilitate the identification of DAMPs and viral ingredients by the immune system, which induces the release of type I interferons. Type I interferon production induces a comprehensive immune response to attack the tumor. In addition, T cells may also be activated by the STING pathway, which enhances the immune response to the tumor [23,37,62].

## 4. Design and Construction of oHSV

**Figure 1:**
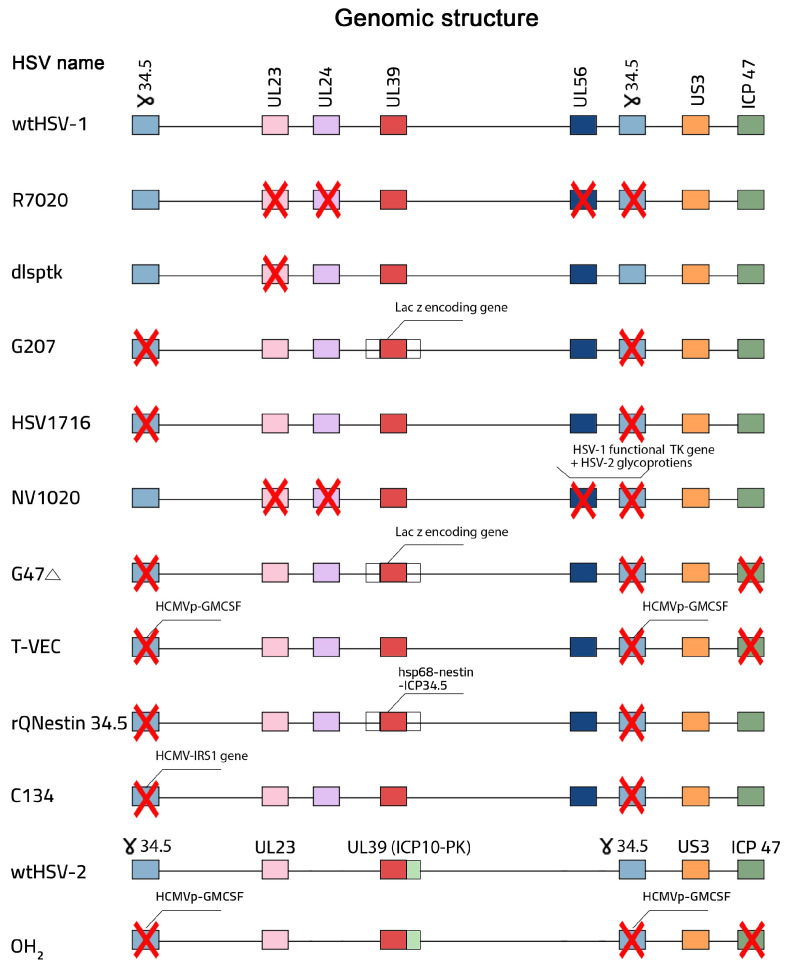
Major recombinant oHSVs that have been developed and tested in preclinical and clinical trials over the last four decades. The red cross represents the corresponding gene knockout or inactivation.

Safety and oncolytic activity are the two most important factors that must be considered during oHSV design to ensure that the virus selectively attacks tumors without affecting normal tissues. Except for spontaneously developing HF10, oHSV was generated by genetic engineering [6]. Recombinant DNA technology has been used to develop oHSV by deleting non-essential viral genes and making engineering modifications in viral glycoproteins to target the tumor-associated surface molecules [6]. From the 1980s to now, many versions of oHSVs have been developed and tested in preclinical and clinical trials (Figure 1) [6]. In 1988, R7020, which is a genetically modified HSV-1, was created. R7020 is characterized by the deletion of the *UL56* gene, one copy of the γ34.5 gene, the *UL24* gene, and the thymidine kinase-encoding gene, which enables it to act as an HSV-1 vaccine in animal models [63,64,65]. NV1020 was later derived from R7020 and acts as an oHSV for the treatment of cancer [63,66]. Furthermore, in 1989, dlsptk was created by the deletion of the *TK* gene. This deletion prevented dlsptk from infecting non-dividing cells and targeting tumor cells [39,45,67,68]. Around the end of the 1990s, two oHSVs were created: G207 and HSV1716. Both of them are characterized by the deletion of the *ICP34.5*-encoding gene, but G207 has an additional modification: *LacZ* gene insertion into the *ICP6* gene locus [6,69,70,71,72,73].

These modifications enable both of these oHSVs to selectively target cancer cells and minimize the damage to normal cells. In the early 2000s, many different oHSVs were created [6]. Around 2001, G47Δ was created by applying one additional modification to the G207 genome. G47Δ has three genetic modifications: deletion of both the α*47* and *ICP34.5*-encoding genes and *LacZ* gene insertion into the *ICP6* gene locus. The deletion of the α*47* gene allows the virus to enhance the activation of the immune system against tumor cells, becoming less effective on normal cells [6,55,74,75]. Around 2005, OncoVEX^GM-CSF^, rQNestin34.5, and C134 were created [5]. OncoVEX^GM-CSF^ was developed by BioVex, which is a biotechnology company, and is characterized by the deletion of the *ICP34.5*- and *ICP47*-encoding genes and insertion of the *GM-CSF* gene [76,77]; rQNestin34.5 is characterized by the reinsertion of one copy of *ICP34.5* within the genetic material of the virus under the influence of a specific promoter called hsp68 and a specific enhancer called the nestin gene [69,78,79]; furthermore, C134 is an oHSV created by combining specific genes from two viruses, and its basic version features deletion of the *ICP34.5* gene in combination with the *IRS1* gene from the human cytomegalovirus [80,81]. Around 2010, M032, which is an armed oHSV, was created by the incorporation of the human interleukin 12-producing gene. This combination allows the virus to stimulate the immune system against tumor cells [82]. Between 2015 and now, RP1, RP2, RP3, ONCR-177, and OH2 have been created and tested in clinical trials [6,83].

## 5. Preclinical and Clinical Trials of oHSVs

### 5.1. Talimogene Laherparevec (T-VEC, or Imlygic)

The first oncolytic viral therapy, which was authorized by the FDA for treating advanced unresectable melanoma, is T-VEC. T-VEC is characterized by the deletion of specific infected cell protein-encoding genes known as the *ICP34.5*- and *ICP47*-encoding genes [84]. These deletions enable T-VEC to improve antigen presentation through the transporter associated with antigen processing (TAP) machinery while limiting viral replication in cancer cells [84]. In 2003, a preclinical study, which included in vivo mouse models and different tumor cell lines, was conducted by Biovex to test T-VEC for treating cancer [77]. T-VEC showed a high efficacy in killing different human tumor cell lines. In addition, notable shrinkage and clearance were observed in injected and non-injected tumors in the in vivo mouse models, particularly for A20 lymphoma, as a result of T-VEC treatment. It was believed that the decrease in the size of non-injected tumors resulted from GM-CSF’s effectiveness with regard to antitumor immunity [77]. In another study, T-VEC showed tumor regression in mouse models with A20 lymphoma, with a rate of 70–100% in injected tumors and 50–60% in contralateral tumors [85]. Furthermore, in another study, T-VEC injection into mice with mNectin-B16F10 melanoma resulted in effective tumor growth inhibition and better survival rates [86].

Preclinical models have also evaluated the effectiveness of combining T-VEC with other immunotherapies, such as checkpoint blocking, and have opened the door for this new therapeutic paradigm in humans [84]. A phase 1 clinical study, which included 30 patients suffering from different cancer types such as breast, colorectal, melanoma, and head and neck cancer, showed that T-VEC was commonly tolerated. Some side effects, such as local inflammation and fever, were observed, especially in patients with negative HSV [87]. Additionally, a phase II clinical study, which included 50 patients suffering from unresectable metastatic melanoma (20% of cases were stage IIIc and 80% of cases were stage IV), showed an overall response rate of 26% [88].

The key outcomes of this study were that complete regression was observed in eight cases, in addition to 10% of cases with partial regression. Flu-like symptoms were observed as a side effect of T-VEC [88]. A phase III clinical study focused on comparing the efficacy of T-VEC and GM-CSF on unresected stage IIIB to IV melanoma [89]. The outcomes demonstrated that T-VEC showed higher effectiveness, which was represented by a durable response rate of 16.3% and an overall response rate of 26.4%, while GM-CSF showed lower effectiveness, which was represented by a 2.1% DRR and a 5.7% ORR. Additionally, when compared to GM-CSF therapy, the overall survival (OS) of treated cases was significantly improved with T-VEC, and, despite some side effects such as fatigue and pyrexia, in general, T-VEC was safely tolerated [89].

### 5.2. Delytact (Teserpaturev, or G47Δ)

The first brain cancer oncolytic viral therapy (OVT), Delytact, has been approved; it was developed from the HSV-1 (F) strain. G207 is the original virus from which G47Δ was engineered by applying an extra mutation, specifically *ICP47* gene deletion, to the genetic material. G47Δ also contains two other mutations: deletion of the *ICP34.5*-encoding gene and *ICP6*-encoding gene inactivation [84]. Preclinical studies have proven the effectiveness of G47Δ by observing OVT through two distinct mechanisms: direct oncolytic viral replication and antitumor immunity activation. It was proven that G47Δ showed a higher oncolytic and antitumor activity than G207, in addition to having great safety features [84]. In Japan, a phase II clinical study at the University of Tokyo, which involved 19 patients who had previously undergone different treatments, was conducted to examine the efficacy of G47Δ in treating recurrent glioblastoma. The key outcomes of this study were that about 84.2% of cases survived for about 12 months after receiving G47Δ [90]. During this study, only one case responded partially to G47Δ treatment, while in the other cases, the disease remained stable. Furthermore, biopsy analysis showed that the level of tumor immune cells, particularly CD4^+^ and CD8^+^, increased, suggesting that antitumor immunity activation was elicited as a treatment response. Although there were some side effects, such as fever and nausea, G47Δ treatment was commonly tolerated. G47Δ was approved as the first oncolytic viral therapy for glioblastoma in Japan following the favorable results of this study [90].

### 5.3. HSV1716 (Seprehvir)

HSV1716 belongs to HSV-1 strain 17 and is characterized by *ICP34.5*-encoding gene deletion; its effectiveness has been tested through many preclinical and clinical studies [84,91]. A preclinical study for treating metastatic breast cancer in 4T1 cells showed that HSV1716 achieved limited replication in 4T1 cells, which had a slight impact on tumor growth. Mice receiving intratumoral injections of HSV1716 demonstrated a reduction in the growth of 4T1 tumors, which led to longer survival periods [92]. Additionally, HSV1716 had a large impact, limiting metastatic growth in the injected mouse lungs. Analysis of tumors after HSV-1716 therapy highlighted the existence of CD4^+^ and CD8^+^ T cells, which proved that HSV-1716 played a great role in activating the antitumor immune response [92]. Furthermore, HSV-1716 had a positive effect on the tumor microenvironment through activating different types of T cells. HSV1716 injection in a female host M3-9-M model induced intratumoral CD4^+^ and CD8^+^ T cells; additionally, the combination of HSV1716 with anti-PD-1 therapy showed a higher activation of these T cells, resulting in better therapeutic effects in this model [93]. Additionally, HSV-1716 achieved good effects as a myeloma target in murine models, especially in combination with bortezomib [94]. Many clinical trials showed that HSV-1716 was generally safe and effective for different types of tumors, such as melanoma, mesothelioma, and high-grade glioma [84].

A phase I clinical trial has been conducted to determine the safety and antitumor immune activity of HSV1716 in nine young patients with refractory extracranial cancers. These patients received HSV1716 through intratumoral injection. HSV-1 viral genetic material was detected through PCR analysis, and an antiviral immune response was also detected. However, there were some side effects, such as low-grade fever and mild cytopenia; there were no dose-limiting toxicities. In general, this study showed that HSV1716 was commonly tolerated. The promising results from preclinical and clinical studies show the need for future research on HSV1716 to improve its oncolytic activity and to find out more about combination therapies [91].

## 6. Combining oHSV with Other Therapies

Oncolytic HSV vectors have been designed to selectively attack actively proliferating tumor cells, similar to other traditional cancer treatments such as chemotherapy and radiotherapy. Unfortunately, these classic therapeutic approaches are associated with a narrow therapeutic index and often have very serious toxicities. Additionally, the genetic heterogeneity of tumors can also lead to the selection of phenotypes resistant to single-agent therapies. In contrast, oHSV vectors have a different mechanism of action that remains effective in tumors resistant to chemotherapy and radiation. Often, the effectiveness of replicating viruses increases with time, while chemotherapeutic drugs usually act less effectively [25]. Furthermore, many studies have shown that the combination of oHSV vectors and other classic therapeutic approaches is a promising strategy that results in an improved cancer treatment efficacy and overcomes the limitations associated with the classic modalities in treating cancer [25,95]. Recently, immunotherapeutic agents such as immune checkpoint, histone deacetylase (HDAC), MAPK pathway, and TGFβ inhibitors have been used in combination with oHSVs, inducing an antitumor immune response and achieving a higher efficacy in targeted cancer cells, as shown in Table 1 [6,96,97,98].

## 7. Quality Control and Evaluation Principles of Oncolytic Drugs

**Figure 2:**
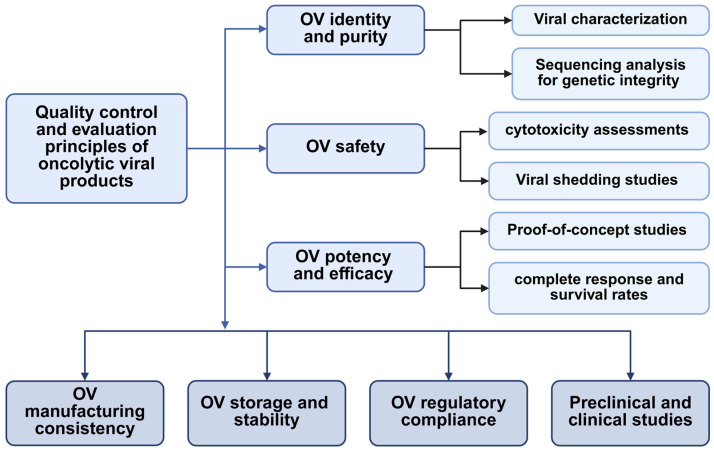
The basic principles for the quality control and evaluation of oncolytic virus products.

Biological product licensing, including oncolytic virus products, requires showing that the products are “safe, pure, and potent”. Quality control and evaluation principles of oncolytic viruses are essential for validating and improving the efficacy, safety, and consistency of OVs for therapeutic application. Oncolytic viral products are derived from infectious viruses via specific genetic modifications that lead to viral attenuation. These modifications cause the oncolytic viruses to be less toxic or infectious than the original strain, but there is still a possibility that the oncolytic viruses may be transmitted during the modification process as infectious intact viruses. This possibility highlights safety concerns connected to the risk of transmission to the surrounding environment. To understand this risk, many of the oncolytic quality control principles that are applied for the target patients may be applied before approval [112,113]. The quality control and evaluation of oncolytic viruses require a comprehensive strategy that involves identity, purity, and safety testing, stability studies, risk management, manufacturing consistency, and regulatory compliance (Figure 2). Analytical methods used to assess the quality of OV products should provide an evaluation of assay performance characteristics (i.e., accuracy, sensitivity, and specificity) to justify that the method is fit for purpose. These key concepts confirm the effectiveness, safety, and consistency of OVs for therapeutic application through preclinical and clinical studies [28,114].

### 7.1. OV Identification and Purification

Until now, the oncolytic viral research field has lacked specific standards and guidelines for OV manufacturing, resulting in distinct obstacles during the production and profiling of OVs. However, gene therapy guidelines should be followed during OV agent production to achieve complete profiling and characterization of the oncolytic virus and confirm the OV reliability. To design safe and effective OV products, clear and identified acceptance criteria and process ranges should be established. Product quality testing guarantees that the product is of acceptable quality (safety, identity, purity, and potency). OVs are identified through intensive viral characterization and molecular viral analysis, focusing on uncovering any genotypic or phenotypic modifications that are applied during viral design to improve their efficiency, safety, and selectivity in targeting cancer cells [28,29,114]. Tests for identity, sterility, and endotoxins should be performed on formulated product in the final container to ensure that product mix-ups and microbial contamination do not occur. Furthermore, purity testing is important to confirm that the OV product is free from any contaminants. This examines the presence or absence of replication-competent viruses to confirm that oncolytic viral products are not infectious agents. Additionally, sequencing analysis methods must be performed during the characterization process to ensure the oncolytic viral genetic integrity [28,114].

### 7.2. OV Safety

The oncolytic viral safety is determined during preclinical studies, in which cytotoxicity assessments related to therapeutic applications are performed. To understand and explain safety profiles, in vitro cytotoxicity assays must be conducted [28,114,115]. Tests performed to assure OV product safety, including microbial testing, should have sufficient performance. Notably, to assure the safety of OV products, the producer should qualify the assays used to determine the dose (e.g., viral vector genome titer by qPCR, multiplicity of infection (MOI), plaque-forming units) prior to initiating potency studies [116]. Additionally, viral shedding studies must be performed to evaluate the transmission risks. To apply the safety principles through clinical trials, the standards from the International Council for Harmonisation of Technical Requirements for Pharmaceuticals for Human Use (ICH) could be followed [28,114,117]. To evaluate viral shedding hazards, biodistribution studies and dose toxicity assessments must be incorporated into these studies [28,114,118,119,120].

Shedding data collection during the design of OVs is an essential process to understand the potential of transmitting OV agents to the surrounding environment. Animal shedding data provide essential information, but they are still unable to clearly indicate human shedding profiles [29,121]. Shedding studies must continue during clinical studies. OV biological features and their administration method are considered to be two of the most essential factors affecting shedding characteristics [113]. Intratumoral administration of T-VEC showed good results in killing cancer cells and treating melanoma. However, T-VEC may spread to different parts of patients’ bodies away from the injection site and into the surrounding environment. It has been proven that when wild-type HSV-1 infects pregnant women, it may pass through the placenta and affect the fetus. Thus, according to T-VEC safety standards, T-VEC is not acceptable for pregnant or low-immunity patients [122,123]. Additionally, OV selectivity must also be tested via in vitro analysis to confirm that OVs selectively infect tumor cells and avoid normal cells. This analysis will allow us to determine the cytotoxicity and replication ability of OVs before starting human clinical trials [29]. Oncolytic viral shedding, biodistribution, and efficacy experiments require an appropriate selection of animal models for preclinical trials [29,113]. In clinical studies, pharmacokinetic studies should focus on quantifying the OV levels in patient bloodstreams by using quantitative nucleic acid amplification techniques such as real-time polymerase chain reaction or by infectivity assays. This process provides essential information about viral proliferation and replication in order to assess the effect of viral shedding on patients’ health and safety. Furthermore, tests to check for previously acquired immunity against OV strains must be performed to confirm the safety of therapeutic strategies [29].

### 7.3. OV Potency and Efficacy

Potency tests, along with other tests, are performed as part of product quality evaluation. Potency measurements are used to determine that only products that meet acceptance criteria are administered during clinical investigations and following market approval. Potency is determined based on individual product attributes. The complexity of OV products can present significant challenges to establishing potency assays. Thus, relevant and meaningful potency measurements need to be developed, and sufficient product characterization data, including molecular, biochemical, immunologic, phenotypic, and biological properties, should be collected [124]. Oncolytic viral potency measurements include estimations of the viral yield-to-infectivity ratio in tumor cells, which are important for evaluating the viral product efficacy. To assess the standard potency of OV agents, in vitro cytotoxic assays and biological characterizations should be conducted in tumor cells. Furthermore, to evaluate OVs’ efficacy, their ability to infect only tumor cells while avoiding normal cells must be determined during preclinical studies, referred to as proof-of-concept (POC) studies. POC studies should provide clinical results such as overall survival rates, complete response rates, and tumor immunity responses that support the OVs’ efficacy and safety before they can be applied in clinical trials [28,114].

### 7.4. OV Manufacturing Consistency

OV manufacturing consistency is an essential measurement in OV quality control that confirms OV quality and uniformity. The OV manufacturing process and process controls should be accurately recorded, including the following: cell culture; cell expansion; virus infection; harvests; purification; filling; storage and shipping conditions. The manufacturing process should be described using a process flow diagram, and process controls and in-process testing (e.g., viability, impurities, titer) should be clearly identified, and operating parameters (e.g., cell passage number, viral stock lots, reagents, temperature, pH, CO_2_, dissolved O_2_, process times) should be acceptable. Process performance parameters should be monitored for consistency, with process trend analysis and process parameter evaluations helping to determine and establish process quality control strategies [125]. The creation of banking systems, such as master cell bank (MBC) and working cell bank (WCB), is essential for sustaining a uniform and reliable source for viral agents. Purity and characterization testing must be applied on the MCB and WCB to confirm that contaminants are absent, validating that the viral particles used for construction are stable, reliable, and suitable for clinical application. Generally, the consistency of OV manufacturing is essential for the reproducibility of this process throughout various batches [28,114].

### 7.5. OV Regulatory Compliance

Health commissions, including the Food and Drug Administration (FDA), the European Medicines Agency (EMA), the National Medical Products Administration (NMPA), etc., establish the standards and guidelines that must be followed to achieve OV regulatory compliance. These standards highlight that there must be a detailed explanation of the reasons for every specific OV design, which must be thoroughly scientifically researched. Additionally, to achieve the required regulatory compliance, preclinical studies must investigate OV quality control measures, such as manufacturing consistency, purity, and biological characterization, that provide proof of the safety and effectiveness of OVs for treating cancer [28,114]. Due to the complexity of OV products, the evaluation principles and analytical methods used to assess the quality of products are not fixed; the safety and potency should be determined based on individual OV product attributes and evaluated on a case-by-case basis.

### 7.6. OV Storage and Stability

As viral modifications may naturally occur in OVs stored in virus banks for future usage, continuous analysis must be performed to ensure viral reliability, effectiveness, and safety. OV product stability should be demonstrated at all stages of product development, and manufacturers should demonstrate that the product is within quality limits for the duration of the planned study. However, an incremental approach may be appropriate for setting acceptance criteria (ACs) to support stability. For example, data to support the stability of the OV product for phase 1 studies can be based on data from nonclinical lots or highly similar product lots that have been stored in the same way (e.g., the same concentration, formulation, container, and storage temperature). Additionally, the stability of genetically modified OVs may be evaluated based on the ICH Q5B guidelines. Genetic stability should be evaluated in the context of the history and selection procedures of wild-type or attenuated OVs [29,114].

### 7.7. Preclinical and Clinical Studies

Preclinical and clinical studies represent the two key stages in which OV quality control principles can be designed and applied to show the proof of principle (POP) for oncolytic viral agents in treating cancer. Shedding, purity, biodistribution, pharmacokinetics, and identity studies must be conducted in preclinical studies to prove the effectiveness and safety of OV products. Additionally, an appropriate animal model must be selected, because the therapeutic results could be affected by a species’ sensitivity to viral infection. When selecting an animal species for nonclinical studies, key considerations include whether the OV product is active in the species, the technical feasibility of using the procedure or clinical delivery device for administration, the comparability of the anatomy and physiology between animals and humans, and the sensitivity of the selected species to potential toxicities of the OV product. The biological relevance of a particular animal or disease model to the target patient population should also be considered.

A properly conducted clinical trial should include appropriate blinding and random assignment of subjects to either a treatment or a concurrent control group, which will generally allow determining if the treatment effect is attributed to the product. During clinical studies, OV safety and dosage testing studies are the first concern in the primary stages; however, the advanced phases focus more on estimating the OVs’ therapeutic effectiveness via evaluating the survival rate, objective response rate, complete response rate, best overall response, and immune response. Additionally, OV shedding studies must also be performed during clinical studies to avoid transmission from the treated patients to the public [28,114]. Some clinical trials have demonstrated that oHSVs could cause HSV infection, tachycardia, pleural effusion, severe hypotension, and seizures [87,91,122,126,127]. Preclinical studies have shown that HSV1716 is well-tolerated through direct intratumoral administration in animals and cells [91]; however, other clinical trials showed that HSV1716 could cause adverse effects, such as headaches and flu-like symptoms, and severe effects such as seizures and urinary tract infections [128]. Thus, some protective measures have been approved for use before oncolytic virotherapy to reduce severe effects. Drinking a large amount of water or injecting with saline was proven to be a successful way to protect patients from hypotension [129,130], and some antiviral drugs, such as acyclovir, can treat and manage HSV infections caused by oHSVs [131,132].

## 8. Limitations and Strategies to Overcome Them

Oncolytic herpes simplex virus therapies represent a promising class of cancer immunotherapy, with products such as talimogene laherparepvec (T-VE/Imlygic^®^, Amgen, Oaks, CA, USA) and Delytact (teserpaturev/G47Δ, Daiichi Sankyo, Japan) already approved in the United States and Japan, respectively. However, oHSV development and approval face regulatory challenges due to their complex biological nature, safety concerns, and manufacturing intricacies. For example, oHSV vectors are often genetically modified (e.g., through the deletion of ICP34.5 and ICP47 to enhance safety and immunogenicity). Regulatory agencies (FDA, EMA, NMPA) require proof of genetic consistency across production batches. OV products have multiple complex active ingredients, and their mechanism of action is not fully characterized. A lack of standardized assays across different OV constructs is one of the challenges. Thus, in many cases, a single biological or analytical assay may not provide an adequate measure of safety and potency. If one assay is not sufficient to measure the product attributes, multiple complementary assays could be used to measure different attributes.

Oncolytic HSVs have limitations that act as a barrier to achieving a favorable therapeutic response. Pre-existing HSV immunity may reduce efficacy, while excessive immune reactions (e.g., cytokine storms) pose safety risks. Oncolytic HSVs are typically grown in Vero cells (African green monkey kidney cells), requiring strict adventitious agent testing. The biological features of oHSV-1 enable it to spread from one cell to another, while limiting its spread through the blood [133]. This has led to oHSV being injected into patients through intratumoral injections, sparing the use of intravenous injections. However, intralesional administration of T-VEC in melanoma cases showed a favorable survival rate and lesion regression, and another study showed that fibrillar collagen in the extracellular matrix acts as a barrier for the oHSV distribution in human melanoma cells, which worsened the therapeutic outcomes [133,134]. Thus, to enhance the oHSV distribution, the extracellular matrix of melanoma cells is injected with bacterial collagenase to destroy the fibrillar collagen, leading to improved therapeutic outcomes [134]. However, γ34.5 mutant oHSVs showed a promising effect in targeting tumor cells, while another study demonstrated that tumor hypoxia reduced the potency of γ34.5 mutant oHSVs [49]. C134, which is a chimeric HSV-1 created by combining specific genes from two viruses (the basic model features the γ34.5 mutant oHSV in combination with the IRS1 gene from human cytomegalovirus), showed higher cytotoxicity and potency than γ34.5 mutant oHSVs under hypoxic conditions [49,135]. However, different versions of oHSVs have led to favorable outcomes in many studies, which have shown that complete tumor clearance is hard to achieve by only using oHSVs [136]. Various aspects, such as the immunosuppressive tumor microenvironment, act as barriers for accomplishing the maximal therapeutic effect of oHSVs [137]. Therefore, the structure of oHSVs can be modified to improve their potency by adding different therapeutic genes, such as immunomodulatory genes, to create new versions of oHSVs called armed oHSVs [138].

## 9. Future Perspectives

At present, more than 200 clinical trials of oncolytic virus therapies have been conducted worldwide. However, the number of globally approved indications for OV products remains limited, and potential patient populations require further clinical research. Challenges and opportunities occur in the processes of development and clinical trial design for OV products, mainly linked to the following aspects:

(1) Screening tumor types and potentially dominant populations suitable for oncolytic virus treatment;

(2) The different curative effects of different administration routes, such as local injection or systemic administration; oHSV products can be administered systemically, and clinical trials related to the intravenous administration of oHSV are currently underway;

(3) Different combination therapy strategies exploring the synergistic effect of OV and other therapies (such as immune checkpoint inhibitors, chemotherapy, and radiotherapy) and formulating a suitable combination therapy scheme;

(4) The biodistribution of oncolytic virus products, for which tumor tissues should be collected for detection. The most commonly used detection method to detect the specific gene sequence of OV is qPCR; other detection methods include the detection of virus infectivity by the cytopathic method and the detection of protein expression via immunohistochemical technology [113];

(5) The analysis of pre-existing immunity to oncolytic viral skeletons, which may be beneficial for deciding the administration route, dose, and frequency. It is recommended that the baseline pre-stored antibodies and the titer of neutralizing antibodies are detected after OV administration, but it is still unclear whether neutralizing antibodies have an impact on the OV efficacy.

Oncolytic herpes simplex viruses are among the most promising approaches under consideration as a cancer therapy due to their distinct biological characteristics, including their large genomic size, which contains 152 kilobases with about 30 kilobases transcribing non-essential genes. This allows for genetic modifications of non-essential genes to provide greater specificity and efficacy in the infection and killing of tumor cells while avoiding normal tissue damage. *ICP34.5*-encoding gene deletion improves the safety of oHSVs through limiting neurovirulence but preserves their oncolytic activity. Additionally, oHSVs’ dual mechanism of action, through combining the two strategies of direct tumor lysis and host immune response activation, could therefore represent an exciting way of further improving their antitumor efficacy. Indeed, several clinical trials and approvals involving therapies such as T-VEC and Delytact have proven that oHSVs are capable of killing tumor cells and eliciting an immune response to malignancies. Despite the achievements made so far, much remains to be investigated in the optimization of the design of oHSVs and, therefore, their applications: their safety profile should be improved, combination therapies should be established, and patients should be predictively selected based on biomarker testing. Future studies should be aimed at refining genetic modifications for enhanced specificity and efficacy, exploring new combination strategies with immunotherapies and targeted agents, and addressing complexities in tumor microenvironments. In summary, ongoing advances in oHSV research hold great promise for a revolution in cancer treatment, with hopefully more effective and durable therapeutic options for patients.

## Figures and Tables

**Table 1:** Summary of the studies focused on the combination of oHSV with immunotherapeutic agents that have achieved promising results.

oHSV	Combination Therapy	Type of Cancer	Results
	**Immune checkpoint inhibitors**		
T-VEC	Ipilimumab	Advanced melanoma	T-VEC plus ipilimumab vs. ipilimumab alone: ORR (39% vs. 18%); 52% of the cases exhibited responses in non-injected visceral lesions [99,100].
T-VEC	Pembrolizumab	Advanced melanoma	CRR: 33% with an increase in CD8+ TILs and PD-L1/IFNγ after receiving T-VEC [101].
OncoVEXmGM-CSF (mT-VEC)	Anti-CTLA-4	In vivo mouse tumor models	Combination therapy increased the survival period vs. that of single treatment. Improved antitumor immunity (tumor-specific splenocytes) [102].
HF10	Ipilimumab	Advanced melanoma	This combination achieved a BOR of 41% in a phase II clinical trial, which means nearly half of the patients responded [103].
OH2 (T-VEC-like oHSV-2)	Anti-PD-1 (HX008)	Advanced solid tumors	Durable iPR was observed in the patients either receiving OH2 or in combination with anti-PD-1 (HX008) [104].
HSV1716	Anti-PD-1	Rhabdomyosarcoma syngeneic mouse models	MHC I controlled the rate of response, as tumors with an elevated expression of MHC I responded to the therapy, while the therapy did not affect tumors with a low expression of MHC I [93].
G47D-mIL12	Anti-PD-1 and anti-CTLA-4	GBM	Most mice responded to this triple combination therapy. High expression of macrophages, but low expression of CD4^+^ Tregs [105].
	**Histone deacetylase (HDAC) inhibitors**		
OncoVEXmGM-CSF (mT-VEC)	Valproic acid	Melanoma	Antitumor immunity was activated by enhancing the expression of IFN, NK cells, and DCs. Virus replication was increased in melanoma cells [106].
	**MAPK pathway inhibitors**		
mT-VEC	Trametinib	BRAFV600E melanoma	Tumor growth inhibition and antitumor immunity activation; 40% of the injected mice showed tumor elimination, while 70% of the treated mice showed rechallenge protection [107].
mRP1	PLX4720	Thyroid tumor cell lines with BRAFV600E	Antitumor immunity activation and tumor growth regression [108].
	**TGFβ inhibitors**		
HSV1716	A8301	Rhabdomyosarcoma	Antitumor immunity activation and increased survival period. A complete durable response was observed in 20% [109].
rQNestin34.5	ID11	Glioblastoma	Prolonged survival period [110].
oHSV MG18L	Galunisertib	Human GBM stem-like cells	A total of 60% were cured, and a prolonged survival period was noted [111].

Abbreviations: ORR, objective response rate; CRR, complete response rate; BOR, best overall response; iPR, immune partial response; GBM, glioblastoma multiforme.

## Data Availability

All data are available from the corresponding author upon reasonable request.
